# Electricity as an Anæsthetic Agent in Dental Surgery

**Published:** 1859-08

**Authors:** 


ELECTRICITY AS AN ANÆSTHETIC AGENT IN DENTAL SURGERY.

At a meeting of the College of Dentists, on Tuesday last, at the board-room in Cavendish square, the report of the committhe appointed for testing the power of electricity in alleviating the pain caused by "tooth drawing" was read by Dr. Richardson. The following is an abstract of the document:

"Sixty-eight cases of extraction were recorded, in sixty-five of which the anæsthetic value of electricity was tested; in fifty-five of those the intermittent current was employed, and in ten the continuous. In these experiments every possible modification was introduced. The poles of the batteries were reversed in different cases; the force of the current, as indicated by the sensations of the patients, was varied; and every necessary precaution was made to secure insulation of the operator. In a large proportion of these cases the results were negative. In some the application of the current produced additional pain; in others, less pain was produced; and, in five cases, there was direct evidence of relief.

"In cases where relief was most apparent, the committee believed that the insensibility was general, the patient being at the time of the operation in a state of syncope. In cases where it was expressed that the pain was less than had been experienced on previous occasions when no electricity was used, the committee consider that the apparent benefit was traceable to four causes—diversion of sensation, less difficult in extraction, syncope more or less marked, and differences in method of operating.

"Cases in which pain was increased by the current were those of recent inflammation of the periosteum, or where abscess was present. In regard to the direction of the current of electricity, its force as computed by the sensations of the patient, the position of the poles, and the different forms of electrical apparatus and currents, the committee could arrive at no affirmative results; differences in these respects indicating in the main no specific differences in effect.

"In a final point the committee were unanimous, that in not one instance did any member observe the nearest approach to local anæsthesia. At the same time, the members were of opinion that the intermittent current was allowable in certain cases as a means of producing a diversion of sensation. But as, in a scientific point of view, the electrical current could not be accepted as an anæsthetie, the committee had no data on which to recommend any special electrical apparatus, nor any particular method of applying electricity in dental operations."

